# Perioperative Morbidity and Mortality of Laparoscopic Sleeve Gastrectomy (LSG) in a Single-Surgeon Experience on 892 Patients Over 11 Years

**DOI:** 10.1007/s00268-023-07123-0

**Published:** 2023-08-07

**Authors:** Mohamed Abdul Moneim Amin El Masry, Islam Abdul Rahman

**Affiliations:** 1 General Surgery, Faculty of Medicine, Cairo University, Cairo, Egypt; 2 General Surgery, Military Production Specialized Medical Centre, Cairo, Egypt

## Abstract

**Background:**

Laparoscopic sleeve gastrectomy (LSG) has been the most frequently performed bariatric procedure since 2014, with continually growing popularity. This study aimed to present our 30-day morbidity and mortality following LSG over a period of 11 years.

**Patients and methods:**

This is a retrospective study that was based on prospectively collected data from patients undergoing LSG by the same surgeon from July 2011 to the end of August 2022. The LSG-associated 30-day morbidity and mortality and the risk factors for 30-day morbidity were assessed.

**Results:**

This study included 892 patients who underwent LSG over the course of 11 years. Early postoperative adverse events were encountered in 16 patients (1.79%). Overall, twelve patients (1.35%) required blood transfusions, and two patients (0.22%) required ICU admission. The re-operation rate was 0.9% (*n* = 8) and the mortality rate was 0.22% (*n* = 2). The patient’s BMI, hypertension, and revisional surgery were marginally significant/significant predictors of early postoperative morbidity. The mean EBWL% was 63.8 ± 15.55 at the 6-month follow-up.

**Conclusion:**

This study confirms the previously reported LSG's short-term safety in terms of a low rate of 30-day postoperative morbidity and mortality. Preoperative BMI, hypertension, and revisional surgery are risk factors for 30-day morbidity and mortality.

## Introduction

Obesity is currently widely prevalent and considered a pandemic that is conferring several health burdens on human life [1, 2]. Bariatric surgery has shown definite success in the loss of weight and remission of obesity-associated comorbidities in patients who have failed to sustain weight loss by non-surgical approaches [3, 4].

Laparoscopic sleeve gastrectomy (LSG) is a bariatric procedure that has become widely popular owing to its technical simplicity, safety, and efficacy [5]. As per the 2018 IFSO survey, it has been the most frequently performed bariatric procedure since 2014 [6].

Like any surgical intervention, LSG could be complicated by adverse events that may result in mortality. The 30-day morbidity and mortality rate has been recognized as a measure of the safety of a surgical procedure for decades [7]. Identifying the risk factors for LSG 30-day morbidity and mortality would be beneficial when enacting strategies for the perioperative management of the patients undergoing LSG, with particular concern for the vulnerable groups.

To the best of our knowledge, this is the first study published from Egypt based on a single surgeon's experience over more than 10 years in LSG to present our 30-day morbidity and mortality following LSG.

## Patients and methods

This study was based on prospectively collected data from consecutively recruited patients who underwent LSG in our institution over a period of 11 years by the first author. The approval of the Research Ethics Committee was obtained before the initiation of the study, and the Declaration of Helsinki was followed.

Patients eligibility for bariatric surgery was based on the criteria for surgical intervention proposed by the NIH consensus panel in 1991 [8] and established by the international medical and surgical societies: the International Federation for the Surgery of Obesity (IFSO), the International Federation for the Surgery of Obesity-European Chapter (IFSO-EC), and the European Association for the Study of Obesity (EASO)) [9–11].

The study patients underwent LSG based on their preference after discussing with the surgeon and presenting the surgical choices. The patients underwent routine preoperative work-up, including dedicated history taking, multidisciplinary clinical assessment, laboratory investigations, and upper gastrointestinal (GIT) endoscopy. Patients with severe gastroesophageal reflux disease (GERD), based on clinical presentation and/or endoscopic assessment, and those with large hiatus hernias were not candidates for LSG. Written informed consent was obtained from the included patients before surgery.

Patients with no available follow-up data on the hospital registry system were excluded. A total of 268 patients who were recruited for bariatric surgery were not included in the study either due to selection of another surgery type or due to ineligibility for LSG.

The surgery was performed as previously established [12]. Briefly, after the standardized preoperative preparation, the surgery was performed under general anesthesia. Pneumoperitoneum was induced, and the sleeve was performed over a 36-Fr bougie with resection from the His angle to approximately 3–4 cm proximal to the pylorus. After surgery, routine postoperative care was provided. The patients were encouraged for early mobilization and received the postoperative diet and supplementation regimen and the schedule of follow-up visits. They were informed to seek medical advice in the event of any adverse event.

Data concerning the patients’ demographics, operative details, and perioperative events were recorded and analyzed.

The 30-day postoperative data were available for all patients (100%). The 6-month follow-up data were available for 890/892 patients (99.8%), after the exclusion of two mortality cases. At the 1-year follow-up, data on 860 patients (96.5%) were available.

### Study outcomes

The primary study outcomes were LSG-associated 30-day morbidity and mortality and the risk factors for 30-day morbidity. The secondary outcomes were the predictors of 30-day morbidity and mortality.

### Statistical analysis

The patients’ data were analyzed with the SPSS statistical software (IBM Corp., Armonk, NY, USA), version 28. Numerical data were expressed as mean, standard deviation, and range. Categorical values were presented as frequencies and percentages, and binary logistic regression analysis was performed to assess risk factors for early postoperative morbidity. A p-value less than 0.05 was considered statistically significant.

## Results

This study included 892 patients who underwent LSG from July 2011 to August 2022 by the same surgeon. The patients had a mean age of 35.98 ± 10.25 years, with females more prevalent (71.9%, *n* = 641). The preoperative weight ranged from 80 to 270 kg, with a mean of 131.39 ± 25.26, the preoperative BMI ranged from 35.7 to 102 kg/M^2^, with a mean of 47.43 ± 7.57 kg/M^2^, and the preoperative excess body weight (EBW) ranged from 35.7 to 102 kg, with a mean of 71.1 ± 5.46. The patients’ comorbidities were dyslipidemia, hypertension, type 2 diabetes mellitus, and obstructive sleep apnea (Table 1).

**Table 1:** Baseline demographic data of the study patients

	Study patients (*n* = 892)	
	Mean ± SD	Range
Age (year)	35.98 ± 10.25	18–60
Baseline weight (Kg)	131.39 ± 25.26	80–270
Baseline BMI (Kg/m^2^)	47.43 ± 7.57	35.7–102

	Count	%
Sex		
Male	251	28.1%
Female	641	71.9%
Comorbidities		
Type 2 diabetes mellitus	90	10.1%
Hypertension	158	17.7%
Dyslipidemia	302	33.86%
Obstructive sleep apnea	13	1.45%

LSG was performed as a primary procedure in 880 patients (98.65%), and as revisional surgery in 12 patients (1.35%). These 12 patients had underwent previous vertical banded gastroplasty (*n* = 6), gastric banding (*n* = 3), gastric plication (*n* = 2), sleeve gastrectomy (*n* = 1). Concurrent cholecystectomy was performed in 72 patients (8.07%) (Table 2).

**Table 2:** Perioperative data of the study patients

	Study patients (*n* = 892)	
	Mean ± SD	Range
Total surgery time (min.)	66.08 ± 21.52	50–110
Hospital stay (days)	1.094 ± 1.15	1–28

	Count	%
LSG		
Primary procedure	880	98.65%
Revisional surgery	12	1.35%
Concurrent cholecystectomy		
Yes	72	8.07%
No	829	91.93%
Drain insertion		
Yes	402	45.1%
No	409	54.9%
Patients-controlled analgesia		
Yes	519	58.2%
No	373	41.8%
Early postoperative adverse events		
Yes	16	1.79%
No	876	98.21%
Required blood transfusion		
Yes	12	1.35%
No	880	98.65%
Required ICU admission		
Yes	2	0.22%
No	890	99.78%
Re-operation rate		
Yes	8	0.9%
No	884	99.1%
Mortality rate		
Yes	2	0.22%
No	890	99.78%

Prior to October 2016, early ambulation and lower limb compression with stockings were used for DVT prophylaxis (464 patients; 52.02%). After then, one week of anticoagulant administration was adopted in addition to the previous measures for DVT prophylaxis (428 patients; 47.98%).

During surgery, drain insertion was indicated in 402 patients (45.1%). The total surgery time ranged from 50 to 110 min, with a mean of 66.08 ± 21.52 (Table 2).

Since January 2016, patient-controlled analgesia was implemented for all patients (519 patients; 58.2%) (Table 2).

Early postoperative adverse events were encountered in 16 patients (1.79%). The postoperative complications were encountered before discharge in 10 patients and after discharge and during the first 30 days after surgery in six patients. The total hospital stay ranged from 1 to 28 days (Table 2).

Six patients had intra-abdominal bleeding. Three of them were treated conservatively (two patients received packed RBCs and one required drain insertion and fresh blood transfusion), and three patients indicated packed RBCs transfusion, re-operation, and hematoma drainage, of whom one underwent laparotomy and two underwent laparoscopy.

One patient had a wound hematoma and clinically suspected leakage. The patient was managed conservatively and received fresh fresh-frozen plasma and packed RBCs.

Three patients had intraoperative bleeding and leakage. They were re-operated with, fluid and hematoma drainage, stent placement, and packed RBCs transfusion (laparotomy in one patient and laparoscopy in two patients).

One patient had intra-abdominal leakage that was complicated by abscess formation and was subjected to laparoscopic exploration, fluid drainage, and stent placement under the umbrella of antibiotic therapy.

One patient had a wound hematoma and clinically suspected leakage. The patient was managed conservatively and received fresh frozen plasma and packed RBCs.

There were another two cases of wound hematoma that were managed conservatively. One of them received packed RBCs.

One patient had wound bleeding, which was managed by percutaneous drain insertion.

A massive pulmonary embolism occurred in one male patient, aged 30 years, with a BMI of 43.1 kg/M^2^, hypertension, and dyslipidemia. The patient underwent preoperative thromboprophylaxis through stoking. (The preoperative prophylaxis with one week of anticoagulant administration has not been adopted yet.) The patient was discharged from the hospital after being fit for discharge. After 3 days, the patient was re-admitted with dyspnea and chest pain and was admitted to the ICU where he was managed by anticoagulant and antithrombotic therapy. The patient died in the ICU.

The early postoperative adverse events are summarized in Tables 3 and 4.

**Table 3:** Baseline demographic data of the patients with early postoperative morbidity

	Age (year)	Sex	Comorbidity	Baseline BMI (kg/m^2^)	Surgery year
Case 1	37	Female	None	43.2	2011
Case 2	38	Female	None	44.4	2012
Case 3	25	Male	None	44.2	2013
Case 4	31	Female	None	47.1	2013
Case 5	48	Female	Dyslipidemia	61.9	2013
Case 6	32	Male	Hypertension, dyslipidemia	63.8	2014
Case 7	50.1	Male	None	50.4	2014
Case 8	39	Male	Hypertension, dyslipidemia	57.7	2014
Case 9	30	Male	Hypertension, dyslipidemia	43.1	2015
Case 10	34	Female	Hypertension, T2DM, dyslipidemia	51.1	2016
Case 11	45	Female	None	46.4	2017
Case 12	50	Female	Hypertension, dyslipidemia	63.3	2017
Case 13	29	Female	None	50.6	2018
Case 14	34	Female	Dyslipidemia	63.7	2018
Case 15	36	Female	None	43	2019
Case 16	34	Female	Hypertension, T2DM, dyslipidemia	44.4	2020

**Table 4:** Early postoperative events of the patients with early postoperative morbidity

	Surgery time (minutes)	Adverse events	Management	LOS (days)	Mortality
Case 1	63	Intra-abdominal bleeding	Open exploration	3	No
Case 2	70	Intra-abdominal bleeding and Leakage	Open exploration	28	No
Case 3	71	Intra-abdominal bleeding	Laparoscopic exploration	3	No
Case 4	81	Intra-abdominal leakage with abscess formation	Laparoscopic exploration	4	No
Case 5	62	Wound hematoma	Conservative	2	No
Case 6	50	Intra-abdominal bleeding	Laparoscopic exploration	3	No
Case 7	64	Intra-abdominal bleeding	Conservative	2	No
Case 8	62	Wound hematoma	Conservative	2	No
Case 9	37	Massive pulmonary embolism	Anticoagulation therapy, thrombolytic therapy	5	Yes
Case 10	65	Intra-abdominal bleeding	Conservative	2	No
Case 11	52	Intra-abdominal bleeding	Conservative	2	No
Case 12	53	Intra-abdominal leakage	Open exploration	11	Yes
Case 13	52	Intra-abdominal bleeding and Leakage	Laparoscopic exploration	14	No
Case 14	50	Intra-abdominal bleeding and Leakage	Laparoscopic exploration	13	No
Case 15	71	Wound subcutaneous bleeding	Conservative	3	No
Case 16	110	Wound subcutaneous bleeding	Conservative	3	No

Overall, twelve patients (1.35%) required blood transfusion and two patients (0.22%) required ICU admission, the patient who had intra-abdominal leakage complicated with sepsis (Case 12), and the patient who had massive pulmonary embolism (Case 9). These two ICU-admitted patients passed away, denoting a mortality rate of 0.22%. The re-operation rate was 0.9% (*n* = 8) (Table 2).

Binary logistic regression analysis showed that the patient’s BMI (OR = 1.048, CI 0.999–1.1, *p* = 0.051), hypertension (OR = 0.081, CI 0.016–0.403, *p* = 0.002), and re-do surgery (OR = 0.35, CI 0.125–0.977, *p* = 0.045) were marginally significant/significant predictors of the early postoperative morbidity. Multivariate regression analysis demonstrated that a model containing the patients’ BMI, hypertension state, and type of surgery (primary or revisional) was able to correctly classify 98.2% of cases according to the occurrence of early postoperative morbidity with a *p*-value of 0.003.

At the 6-month follow-up, the mean patients’ BMI was 34.61 ± 6.96 kg/M^2^, and the mean EBWL% was 63.8 ± 15.55%. At the 1-year postoperative follow-up, the mean patients’ BMI at the 1-year follow-up was 29.76 ± 5.75 kg/M^2^ and the mean EBWL% was 84.57 ± 18.41%. Concerning the associated comorbidities, complete resolution occurred in 97.02% of patients with dyslipidemia (*n* = 293), 65.82% of patients with hypertension (*n* = 104), 61.11% of patients with diabetes mellitus (*n* = 55), and 100% of patients with obstructive sleep apnea (*n* = 13). There was an improvement in 27 patients with hypertension (17.09%) and in 11 patients with diabetes mellitus (12.22%).

## Discussion

Despite the reported safety of bariatric surgery, variable rates of perioperative complications were previously reported [13–15]. In this retrospective cohort study that included 892 patients who underwent LSG by a single surgeon in Egypt, the rates of early postoperative morbidity, re-operation, ICU admission, and mortality were 1.79%, 0.9%, 0.22%, and 0.22%, respectively. These figures denote the relative safety of LSG in patients with obesity. Reports of 30-day post-bariatric surgery complications are abundant. In accordance with our findings, the rate of complications in published literature ranged from 1.2 to 7.9% [13], the re-operation rate within 30 days ranged from 0.6 to 1.1% [14, 15], and the mortality rate ranged from 0 to 0.3% [14, 15].

Patients undergoing bariatric surgery are a vulnerable group with an elevated risk of perioperative morbidity [16]. There are still areas for improvement in outcomes after bariatric surgery. The determination of risk factors for early postoperative complications would help optimize the pre- and postoperative patient’s care as much as possible. It is worth noting that in the current study, the two mortality cases did not occur at the surgeon’s initial learning curve, since they occurred in 2015 and 2017 (after 4 and 6 years of the start date of performing LSG for the included patients, respectively), indicating that they were related mainly to the patient’s risk factors rather than the surgeon’s limited experience.

Meanwhile, in the present study, the patient’s BMI, presence of hypertension, and revisional surgery were all predictors of early perioperative morbidity. Both BMI and hypertension are well-established predictors of cardiovascular risk [17]. This could partially explain their association with increased 30-day morbidity and mortality. In congruence with our findings, Elnabil-Mortada et al. implied the patients’ preoperative BMI as a main influencer of early postoperative morbidity [18]. Aminian et al. reported BMI and hypertension as risk factors for post-bariatric early morbidity [19]. DeMaria et al. have validated the Obesity Surgery Mortality Risk Score (OS-MRS) scale. On this scale, 1 point was assigned to each of 5 preoperative variables, including BMI ≥ 50 kg/m2, male gender, arterial hypertension, known risk factors for pulmonary embolism, and age ≥ 45 years. Patients with a total score of 0 to 1 were classified as the lowest risk group, scores 2–3 as the intermediate-risk group, and scores 4 to 5 as the high-risk group [20]. Lak et al. reported metabolic syndrome as a risk factor for post-bariatric surgery morbidity and mortality [21]. Speaking of leakage and bleeding in particular, the study of Aurora et al., which included the analysis of 4888 patients undergoing LSG, reported that there was a significantly higher leak rate in patients with a BMI > 50 kg/m^2^ [22]. The association of hypertension with an increased risk for early bleeding after bariatric surgery has also been reported previously [23].

Concerning revisional bariatric surgery, it has become an essential and necessary adjunct to primary procedures, and with the continuously growing volume of bariatric surgeries, a parallel increase in revisional surgery is mandatory [24]. However, similar to this work, an increased risk of perioperative complications has been linked to revisional bariatric surgery [25–27].

In the current work, the surgery’s short-term efficacy was confirmed by sufficient postoperative weight loss. Furthermore, the present study showed remarkable postoperative amelioration of the associated medical complications. These findings are comparable with several studies, empathizing that LSG provided meaningful weight loss and resolution of obesity-associated comorbidities [28–35].

This study is limited by its retrospective design. However, LSG, based on this study's findings, showed a low rate of early postoperative morbidity and mortality. We believe that a comprehensive preoperative workup might give the surgeon a particular chance to target modifiable risk factors. In our study, this may be applicable by controlling preoperative weight as far as possible, which could further allow some control of the hypertension state since several previous studies have reported that loss of weight is paralleled with clinically significant declines in the sympathetic nervous system activity and renin angiotensin-aldosterone system, which substantially affect blood pressure [36–39]. Observational evidence exists regarding the association between non-surgical weight loss and the control of blood pressure [40]. However, this is to be studied in a further prospective study, including a larger cohort. The surgery's efficacy in inducing sufficient weight loss and improving obesity-associated comorbidities was confirmed in the current study.

## Conclusion

This study confirms the previously reported LSG's short-term safety in terms of a low rate of 30-day postoperative morbidity and mortality. Preoperative BMI, hypertension, and revisional surgery are risk factors for 30-day morbidity and mortality.
